# Characteristics of patients misdiagnosed with Alzheimer’s disease and their medication use: an analysis of the NACC-UDS database

**DOI:** 10.1186/1471-2318-13-137

**Published:** 2013-12-19

**Authors:** Joseph E Gaugler, Haya Ascher-Svanum, David L Roth, Tolulope Fafowora, Andrew Siderowf, Thomas G Beach

**Affiliations:** 1 Center on Aging, School of Nursing, University of Minnesota, Minneapolis, MN, USA; 2 Lilly Research Laboratories, Eli Lilly and Company, Indianapolis, IN, USA; 3 Center on Aging and Health, School of Medicine, Johns Hopkins University, Baltimore, MD, USA; 4 Avid Radiopharmaceuticals Inc, Philadelphia, PA, USA; 5 Banner Sun Health Research Institute, Sun City, AZ, USA

**Keywords:** Alzheimer disease, Diagnosis, Misdiagnosis, Autopsy, Neuropathology

## Abstract

**Background:**

This study compared individuals whose clinical diagnosis of Alzheimer’s disease (AD) matched or did not match neuropathologic results at autopsy on clinical and functional outcomes (cognitive impairment, functional status and neuropsychiatric symptoms). The study also assessed the extent of potentially inappropriate medication use (using potentially unnecessary medications or potentially inappropriate prescribing) among misdiagnosed patients.

**Methods:**

Longitudinal data from the National Alzheimer’s Coordinating Center Uniform Data Set (NACC-UDS, 2005–2010) and corresponding NACC neuropathological data were utilized to compare 88 misdiagnosed and 438 accurately diagnosed patients.

**Results:**

Following adjustment of sociodemographic characteristics, the misdiagnosed were found to have less severe cognitive and functional impairment. However, after statistical adjustment for sociodemographics, dementia severity level, time since onset of cognitive decline and probable AD diagnosis at baseline, the groups significantly differed on only one outcome: the misdiagnosed were less likely to be depressed/dysphoric. Among the misdiagnosed, 18.18% were treated with potentially inappropriate medication. An additional analysis noted this rate could be as high as 67.10%.

**Conclusions:**

Findings highlight the importance of making an accurate AD diagnosis to help reduce unnecessary treatment and increase appropriate therapy. Additional research is needed to demonstrate the link between potentially inappropriate treatment and adverse health outcomes in misdiagnosed AD patients.

## Background

Alzheimer’s disease (AD) is a progressive neurodegenerative disease and is the most common cause of dementia, accounting for about 60% of all cases [1]. The clinical diagnosis of AD is a challenging evaluation process that follows established clinical criteria and requires elimination of other potential causes for dementia [2,3]. Various studies have previously assessed the accuracy of the clinical diagnosis of AD based on autopsy results, or the “gold standard.” A recent and comprehensive study showed that depending on the permissiveness of clinical and neuropathologic criteria, sensitivity ranged from 70.9% to 87.3% and specificity ranged from 44.3% to 70.8% [4]. This and other studies found that between 12% and 23% of patients diagnosed with AD do not have sufficient AD pathology at autopsy to account for the presence of dementia (“misdiagnosed”) [5-9].

The observed misdiagnosis rate may be partly driven by the fact that numerous conditions can mimic symptoms of AD [2]. Some of these conditions constitute other types of progressive dementias (e.g., frontotemporal dementia, vascular dementia and dementia with Lewy bodies) while others may be treatable and possibly reversible conditions (e.g., drug intoxication, depression, nutritional deficiencies, infectious diseases) [10,11]. In a recent retrospective analysis of clinical trials, 63% of deceased patients who were clinically diagnosed with AD while alive were found to have AD with other pathology [12]. In addition, older persons appear to have more etiologies than younger individuals which potentially attenuates the accuracy of clinical AD diagnosis [13]. Other studies have found higher levels of concordance between clinical and post-mortem diagnosis of AD but diminished diagnostic accuracy with other types of dementia [14,15].

Ruling out AD may result in changing patients’ management plans that can lead to further evaluation and testing for the true underlying cause. Excluding AD may also enable appropriate treatment of the true underlying condition. A number of medications have been identified as potentially unnecessary for patients with frontotemporal dementia [16-20] and dementia with Lewy bodies [21,22], whereas treatment with statins, antiplatelet agents and anticoagulants is deemed appropriate for patients with cerebrovascular disease.

At present limited information is available on the clinical, functional and socio-demographic characteristics of persons who have been misdiagnosed with AD based on neuropathologic results [23]. Similarly, it is unknown whether misdiagnosis of AD is associated with potentially unnecessary treatment or may result in patients not receiving treatments that are more appropriate for their conditions. To help address this knowledge gap we expanded on the study by Beach et al. [4] which identified individuals whose clinical diagnosis matched or mismatched their diagnosis per neuropathologic examination post-mortem. Using data collected as part of the National Alzheimer’s Coordinating Center Uniform Data Set (UDS) (NACC-UDS) between 2005 and 2010, Beach and colleagues identified 88 participants misdiagnosed with AD and 438 participants accurately diagnosed with AD [5]. The goal of our study was to address two specific research questions: 1) When compared to accurately diagnosed AD patients, do misdiagnosed patients vary significantly on sociodemographic characteristics, health history, and key clinical and functional outcomes (cognitive impairment, functional status and neuropsychiatric symptoms); and 2) What is the extent of potentially inappropriate medication use among misdiagnosed patients?

## Methods

### Subjects

The National Alzheimer’s Coordinating Center (NACC) [24] serves as the primary repository and data hub of the 34 past and present National Institute on Aging Alzheimer’s Disease Centers (ADCs). Alzheimer’s Disease Centers are located throughout the United States, are based in university medical centers, and are largely in urban areas [4]. Recruitment occurs through referrals from neurologists as well as community outreach efforts. The NACC Uniform Data Set (or NACC-UDS) is a publicly accessible, longitudinal database that includes standardized cognitive, behavioral, and functional data for each ADC participant based on their annual visits. The NACC-UDS was initiated in 2005. Of particular relevance to the current study, the NACC-UDS includes longitudinal data on persons with different etiologies of dementia as well as individuals not diagnosed with dementia. The procedure of AD clinical diagnosis (in living subjects) ranges from that of a consensus panel to a single physician according to each ADC’s diagnostic protocol; however, each ADC generally adheres to standardized clinical criteria as outlined by the DSM-IV or NINDS-ADRDA guidelines [25]. The NACC-UDS provides systematic information on the following domains: demographics, behavioral status, cognitive testing, medical history, family history, clinical impressions, and diagnoses. For more detail on the construction of the NACC-UDS, please see Morris et al. [26]. Ethical approval for the current study was provided by the University of Minnesota Institutional Review Board (IRB#1108E03546).

Participants in the current analysis were previously identified in Beach et al.’s study [4]. NACC-UDS data from 2005–2010 were considered for participants that had at least one UDS assessment, had died, and had brain autopsy results available (n = 1198). Of these individuals, 279 were excluded because they were considered “not demented” during regular UDS assessments or did not have data entered in key diagnostic entry fields (i.e., presence or absence of clinically probable AD and CERAD plaque density or Braak stage) [4].

Of these remaining 919 individuals, two subgroups were the focus of the current analysis. Those in the accurately diagnosed group received a primary clinical diagnosis of probable AD based on NINDS-ADRDA criteria [26] and also received a neuropathological diagnosis/verification of AD per moderate or frequent density on the CERAD neuritic plaque density score [25] and a Braak neurofibrillary tangle stage of III-VI [27]. The classification of those accurately diagnosed with probable AD was based on a comprehensive analysis using various thresholds of CERAD neuritic plaque density score and Braak neurofibrillary tangle stages to find those with the greatest predictive value by Beach and colleagues (n = 438; see p. 268). Those in the misdiagnosed group received a primary clinical diagnosis of probable AD but did not meet the aforementioned neuropathological threshold for AD at autopsy (i.e., a moderate or frequent CERAD density score or a Braak neurofibrillary tangle stage of III-VI; n = 88). The primary neuropathologic diagnoses of those in the misdiagnosed group included primary neuropathologic diagnosis of AD despite a low level of AD histopathology (n = 17), tangle-only dementia or agryophilic grain disease (n = 15), frontotemporal lobar degeneration (n = 15), cerebrovascular disease (n = 10), Lewy body disease, with or without AD (n = 9), hippocampal sclerosis, with or without AD (n = 9), progressive supranuclear palsy (n = 3), corticobasal degeneration (n = 2), neuroaxonal dystrophy/Hallevorden-Spatz-like condition (n = 2), and miscellaneous (n = 6; 1 case each of amyloid angiopathy, “small vessel disease,” “TDP-43 proteinopathy,” limbic encephalitis, Rosenthal fiber encephalopathy, “clinical dementia, no neuropathological substrate”) [4].

In the original Beach et al. analysis, subjects were diagnosed with possible AD (n = 126) using NINDS-ADRDA guidelines [26]. These individuals had a neuritic plaque density average of 2 (SD = 1.2; 0 = none; 1 = sparse; 2 = moderate; and 3 = frequent) and a Braak stage mean of 4.2 (SD = 1.6) at death. Another subgroup of participants (n = 271) was classified as not having either probable or possible AD based on NINDS-ADRDA guidelines, and of these a substantial proportion had a neuropathological diagnosis of AD (n = 107; “false negatives”) or other neuropathological diagnoses: frontotemporal lobar degeneration (n = 60), Lewy body disease with or without AD (n = 31), Creutzfeldt-Jakob disease and other prior encephalopathies (n = 23), progressive supranuclear palsy (n = 18), tangle-only dementia or argyrophilic grain disease (n = 9), corticobasal degeneration (n = 8), Pick’s disease (n = 6), cerebrovascular disease (n = 6), hippocampal sclerosis with or without AD (n = 2), amyotrophic lateral sclerosis (n = 2), and miscellaneous (1 case each of neuronal intermediate filament disease, “leukodystrophy,” and cerebellar atrophy; n = 3). As one of the aims of the current study was to examine whether potentially inappropriate medication use occurred among those who were misdiagnosed as having probable AD [4], those in the possible AD group as well as the false and true negatives in the original Beach et al. analysis were excluded.

### Measures

The following measures available at the first time a probable AD diagnosis was recorded in the NACC-UDS were included. Socio-demographic/background characteristics included age, gender, race (Caucasian vs. non-Caucasian), education (years), marital status (married/living as married or not), and living alone (yes/no). Health history and conditions included any family history of dementia (a parent with dementia, a sibling with dementia and number of other relatives with dementia), health history (any history of cardiovascular disease, cerebrovascular disease, parkinsonian features, other neurologic conditions, medical/metabolic conditions), etiology of AD (AD only or a mixed etiology), and living in a nursing home (yes/no). Since individuals who participated in the NACC-UDS may have received an Alzheimer’s disease diagnosis prior to enrollment (and time of diagnosis prior to NACC-UDS was not recorded), two additional variables were included to address potential heterogeneity of disease stage. A dichotomous variable identified those who were recorded as having or not having a probable AD diagnosis at baseline in the NACC-UDS. Informants also reported on participants’ years since onset of cognitive decline at the initial NACC-UDS visit. Severity of cognitive impairment was measured with the Mini-Mental Status Examination/MMSE (mean score and level of cognitive impairment: normal >24, mild 21–24, moderate 10–20 and severe < =9) [28]. Functional status was assessed with the Functional Assessment Questionnaire/FAQ (total score and proportion of patients with impaired functioning, per total score of 9 or above) [29]. Neuropsychiatric symptomatology was measured with the Neuropsychiatric Inventory Questionnaire/NPI-Q (total score) [30]. Due to extensive missing data on the Geriatric Depression Scale/GDS [31] in the accurately diagnosed group (approximately 30%), depression was assessed using other available measures: a) proportion of depressed subjects who had a “yes” response per the NPI-Q depression or dysphoria item completed by informants of the NACC-UDS participant; and b) severity of depression among subjects identified as depressed/dysphoric on the NPI-Q, also completed by informants.

### Use of potentially inappropriate medications

Data on medication use at or following the first assessment which subjects’ probable AD diagnosis was recorded in the NACC-UDS were considered. The identification of potentially inappropriate medications (the use of potentially unnecessary medications or potentially inappropriate prescribing) was deemed feasible for 3 specific subgroups within the misdiagnosed group based on prior research: those diagnosed post-mortem with either frontotemporal dementia (FTD, n = 18), dementia with Lewy bodies (DLB, n = 9), or cerebrovascular disease (n = 10) [4,18,20,21,32]. Prior to start of the analysis, a neurologist at Eli Lilly and Company created an appropriate and potentially inappropriate medication matrix list based on the NACC-UDS medication checklist. Classification of potentially inappropriate medications was then based on clinical treatment guidelines and available research evidence.

The use of acetylcholinesterase inhibitors was considered potentially inappropriate for subjects whose true diagnosis was FTD at autopsy [20]. A previous study evaluating donepezil in the treatment of FTD relative to matched, untreated FTD patients over six months found that a third of the treated patients experienced increased disinhibited or compulsive acts, which abated with discontinuation of the medication [33]. These and similar observations have prompted a general recommendation to avoid acetylcholinesterase inhibitors in FTD [16,17,33]. Recent randomized controlled trials also suggest that memantine lacks efficacy in the treatment of FTD [34].

The use of antipsychotics, except for quetiapine or clozapine**,** is considered potentially inappropriate for patients with dementia with Lewy bodies (DLB) as these individuals may experience severe side effects or fatal complications if behavioral symptoms are treated with antipsychotic drugs [35]. Patients with DLB are particularly sensitive to developing extrapyramidal symptoms and potentially fatal complications of neuroleptic sensitivity, which affects approximately 50% of DLB patients. Administering antipsychotic medications for behavioral symptoms to patients with DLB can potentially result in serious neuroleptic sensitivity reactions which are associated with significantly increased morbidity and mortality [21,22].

To further assess whether participants in the misdiagnosed group were subject to potentially inappropriate prescribing, we determined the number of participants in the misdiagnosed group with cerebrovascular disease who did not use statins, antiplatelet agents or anticoagulants. These medications are considered appropriate for those with cerebrovascular disease [33]. Thus, the use of potentially inappropriate medication was defined as (a) the use of an anti-dementia drug by those whose true diagnosis was found at autopsy to be FTD, or (b) the use of an antipsychotic drug by those whose true diagnosis was found at autopsy to be DLB, or (c) not being treated with statins, antiplatelet agents or anticoagulants by those whose true diagnosis was found at autopsy to be cerebrovascular disease.

As a sensitivity analysis, the use of potentially inappropriate medications was also assessed using a broader definition which included the above criteria or the use of an anti-dementia drug by any of the misdiagnosed patients (i.e., not confined to those found to have FTD at autopsy). The rationale for broadening the definition was that anti-dementia drugs have an approved indication for the treatment of AD, whereas there is a lack of evidence-based support for non-AD/misdiagnosed individuals receiving such pharmacological intervention [36]. The “off label” use of anti-dementia drugs was, therefore, considered potentially unnecessary for the misdiagnosed subjects in the study. Notably, there is an anti-dementia drug (rivastigmine) that is indicated for dementia due to Parkinson’s disease but none of the misdiagnosed in the current study were diagnosed with this condition.

### Analysis

The principal objective of this analysis was to statistically compare the misdiagnosed and accurately diagnosed groups on sociodemographics, health history, and clinical and functional outcomes (cognitive impairment, functional status, and neuropsychiatric symptoms) as assessed at the first UDS assessment for which a clinically probable AD diagnosis was recorded in the NACC-UDS. Group comparisons for all variables were first conducted using unadjusted bivariate analysis (e.g., unadjusted logistic or multinomial regressions for categorical variables, T-tests for continuous measures). To assess whether the groups significantly differed on key outcomes (severity of cognitive impairment, functional status, and neuropsychiatric symptoms) when their core demographic characteristics, time since onset of cognitive decline and dementia severity were held constant, we conducted two sets of adjusted analyses: one controlling for participants’ key sociodemographic characteristics (age, education, gender, race, and marital status) and the second controlling for the aforementioned sociodemographics as well as time since onset of cognitive decline, a probable AD diagnosis at baseline of the NACC-UDS (yes/no), and dementia severity (categorical, as assessed by MMSE levels noted above). The second adjusted analysis was performed because dementia severity, the presence of probable AD diagnosis at baseline, and time since symptom onset are core clinical characteristics of AD and are correlated with other key clinical and functional outcomes. Analyses of covariance were used for continuous outcomes and logistic or multinomial logistic regression analyses were used for categorical outcomes.

An additional study objective was to examine potentially inappropriate medication use by the misdiagnosed group. Using data on medication use at or following the first assessment when subjects’ probable AD diagnosis was recorded in NACC-UDS, we identified potentially inappropriate medication use for all misdiagnosed participants. SAS version 9.3 [37] was used to extract data and perform all analyses.

## Results

### Sociodemographic background characteristics and health history

Participant socio-demographic characteristics, health history, and bivariate comparisons between the misdiagnosed and accurately diagnosed groups are presented in Table 1. Participants in the misdiagnosed group were significantly (p *<* .05) older than those in the accurately diagnosed group (83.52 years vs. 78.72 years, respectively), were more likely to live alone (17.05% vs. 4.11%, respectively), and were less likely to be married (56.82% vs. 71.00%, respectively) at the time of study entry or first AD diagnosis. For all other socio-demographic characteristics, the two diagnostic groups did not significantly differ. A significantly higher proportion of individuals in the misdiagnosed group had a history of a cardiovascular condition (47.13%) than did those in the accurately diagnosed group (31.49%). Those in the accurately diagnosed group had experienced a significantly longer time since onset of cognitive decline than those in the misdiagnosed group (7.81 vs. 6.30 years, respectively) and were more likely to have a probable AD diagnosis at baseline (94.06% vs. 73.86%, respectively). For all other health history and condition variables the two groups did not significantly differ.

**Table 1:** Socio-demographic and clinical characteristics of the misdiagnosed and accurately diagnosed groups: unadjusted comparisons

	**Misdiagnosed N = 88**	**Accurately diagnosed N = 438**	**Parameter estimate**	**Unadjusted OR (95% CI)**	**p value**
**Sociodemographic characteristics**					
Age, Mean ± SD	83.52 ± 10.31	78.72 ± 10.27	-4.80		<.0001
Years of education, Mean ± SD	14.53 ± 3.56	14.90 ± 3.34	0.37		.3528
Gender, N,% male	52, 59.09	254, 57.99		0.96 (0.60, 1.52)	.8490
Race, N,% minority	5, 5.68	24, 5.48		0.96 (0.36, 2.59)	.9388
*Race*					.
White, N/%	83, 94.32	414, 94.52		(Ref)	
Black, N/%	3, 3.41	17, 3.88		1.14 (0.33, 3.97)	.8414
Asian, N/%	1, 1.14	4, 0.91		0.80 (0.09, 7.27)	.8444
Other, N/%	1, 1.14	3, 0.68		0.60 (0.06, 5.85)	.6614
Unknown, N,%					
Marital status, married/living as married, N,%	50, 56.82	311, 71.00		1.86 (1.16, 2.98)	.0095
Living alone, N,%	15, 17.05	18, 4.11		0.21 (0.10, 0.43)	<.0001
**Health history and conditions**					
Family history of dementia					
Number of “Other demented relatives,” Mean ± SD	0.41 ± 0.80	0.63 ± 1.20	0.22		.1971
Father or mother with dementia, N,%	23, 32.86	183, 47.78		1.87 (1.09, 3.20)	.0225
Any sibling with dementia, N,%	4, 4.55	8, 1.83		0.39 (0.12, 1.33)	.1339
Health history					
Cardiovascular condition, N,%	41, 47.13	137, 31.49		0.52 (0.32, 0.82)	.0055
Cerebrovascular condition N,%	18, 20.69	66, 15.28		0.69 (0.39, 1.24)	.2127
Parkinsonian features, N,%	9, 10.23	39, 8.97		0.86 (0.40, 1.86)	.7087
Other neurologic conditions, N,%	17, 20.00	87, 20.67		1.04 (0.58, 1.86)	.8908
Medical/metabolic conditions, N,%	80, 90.91	361, 82.61		0.48 (0.22, 1.02)	.0574
AD only vs. mixed etiology	56, 63.64	298, 68.04		0.82 (0.51, 1.33)	.4225
Living in nursing home, N,%	13, 14.77	85, 19.41		1.39 (0.74, 2.62)	.3103
Time since onset of cognitive decline (years)	6.30 ± 3.76	7.81 ± 4.01	1.51		.0016
Probable AD diagnosis at baseline	65, 73.86	412, 94.06		5.61 (3.02, 10.41)	<.0001
**Severity of cognitive impairment**					
MMSE, Mean ± SD	19.46 ± 7.66	12.93 ± 9.03	-6.53		<.0001
Normal (>24): N,%	22, 25.00	33, 7.53		1.00 (Ref)	
Mild (21–24): N,%	23, 26.14	67, 15.30		1.94 (0.95, 3.98)	.0699
Moderate (10–20): N,%	24, 27.27	136, 31.05		3.78 (1.89,7.55)	.0002
Severe (<= 9): N,%	11, 12.5	137, 31.28		8.30 (3.67, 18.81)	<.0001
Missing: N,%	8, 9.1	65, 14.84		5.42 (2.18, 13.48)	.0003
**Functional status**					
Functional activities questionnaire (FAQ), total score, Mean ± SD,	20.96 ± 8.52	24.52 ± 7.10	3.55		<.0001
Impaired level of functioning, FAQ > =9, N,%	75, 88.24	405, 95.74		3.0 (1.33, 6.75)	.0080
**Neuropsychiatric symptoms**					
Neuropsychiatric inventory questionnaire (NPI-Q), total score, Mean ± SD	4.84 ± 4.46	6.18 ± 5.35	1.35		.0364
Patients with depression and dysphoria (NPI-Q), N,%	16, 20.25	145, 37.08		2.32 (1.29, 4.17)	.0048
Severity level for patients with depression and dysphoria (NPI-Q), Mean ± SD	1.56 ± 0.73	1.40 ± 0.62	-0.16		.3276

NOTE: CI = Confidence interval, SD = standard deviation, AD = Alzheimer’s disease; MMSE = Mini Mental Status Examination.

### Severity of cognitive impairment and outcomes

Table 1 also provides detail on severity of cognitive impairment, functional status, and neuropsychiatric symptoms (including depression). Individuals in the misdiagnosed group scored significantly (p < .05) higher/better on the MMSE (19.46) than those in the accurately diagnosed group (12.93), with a lower percentage of misdiagnosed participants scoring within the “severe” category (12.5% vs. 31.28%, respectively). The misdiagnosed group also appeared less functionally impaired on the FAQ, on average, and a smaller proportion scored above the impaired clinical threshold than those in the accurately diagnosed group (88.24% vs. 95.74%, respectively). The misdiagnosed also had lower/better average neuropsychiatric scores on the NPI-Q than those in the accurately diagnosed group (4.84 vs. 6.18, respectively). A higher percentage of those in the accurately diagnosed group had depression/dysphoria as measured on the NPI-Q item than those in the misdiagnosed group (37.08% vs. 20.25%, respectively), but the two groups did not differ on the NPI-Q severity of depression indicator.

### Adjusted analyses

We conducted a series of analyses to determine whether the observed group differences in clinical and functional variables were maintained following: a) adjustments for key socio-demographics (age, gender, race, marital status and education); and b) adjustments for key socio-demographics, dementia severity level (MMSE categorical scores), time since onset of cognitive decline, and whether probable AD diagnosis was recorded at baseline (see Table 2). Following adjustments for sociodemographic characteristics only, the results paralleled those of the unadjusted bivariate comparisons; individuals in the misdiagnosed group had significantly (p < .05) higher/better MMSE scores, had significantly lower/better FAQ scores, and were less likely to have depression/dysphoria on the NPI-Q. The NPI-Q total score no longer varied significantly following the adjustment of sociodemographic characteristics.

**Table 2:** Functional and clinical outcomes of the misdiagnosed and accurately diagnosed groups: adjusted comparisons

	**Parameter estimate**	**Adjusted OR**^**a **^**(95% CI)**	**p value**	**Parameter estimate**	**Adjusted OR**^**b **^**(95% CI)**	**p value**
**Severity of cognitive impairment**						
MMSE (Mean)	-5.90		<.0001			
Normal (>24)		1.00 (Ref)				
Mild (21–24)		2.20 (1.04, 4.65)	.0388			
Moderate (10–20)		4.27 (2.07, 8.80)	<.0001			
Severe (<= 9)		8.16 (3.48, 19.15)	<.0001			
Missing		5.93 (2.30, 15.30)	.0002			
**Functional status**						
Functional activities questionnaire (FAQ), total score (Mean)	3.24		.0003	-0.90		.1823
Impaired level of functioning, FAQ > =9		2.56 (1.11, 5.93)	.0281		1.09 (0.40, 2.92)	.8703
**Neuropsychiatric symptoms**						
Neuropsychiatric inventory questionnaire (NPI-Q), total score (Mean)	0.81		.2018	0.55		.4222
Patients with depression and dysphoria (NPI-Q)		1.99 (1.09, 3.63)	.0251		2.15 (1.14, 4.07)	.0183
Severity level for patients with depression and dysphoria (NPI-Q)	-0.18		.2697	-0.21		.2327

NOTE: CI = Confidence interval, SD = standard deviation, MMSE = Mini Mental Status Examination; Analyses of covariance were used for continuous outcomes.

^a^Adjusted for age (continuous – in years), education (continuous – in years), gender, race (white vs. minority), and marital status (married vs. other).

^b^Adjusted for age (continuous – in years), education (continuous – in years), gender, race (white vs. minority), marital status (married vs. other), dementia severity (MMSE), time since onset of cognitive decline, and probable AD diagnosis at Time 1.

When including MMSE, time since onset of cognitive decline and probable AD diagnosis at baseline along with sociodemographics as covariates (see Table 2) the two groups were found to statistically differ on only one outcome: depression. A lower proportion of participants in the misdiagnosed group was found to have depression/dysphoria on the NPI-Q (p = .0183).

We repeated the adjusted models with one sociodemographic variable as an outcome: living alone, as it is apt to have clinical ramifications in our cognitively impaired sample. The misdiagnosed group was more likely to live alone than those in the accurately diagnosed group (OR = .26, 95% CI = .11, .60, *p* = .0018) after adjusting for sociodemographic variables only. However, there was no significant difference in living alone between the misdiagnosed and accurately diagnosed groups after adjusting for sociodemographic variables, MMSE, time since onset of cognitive decline and probable AD diagnosis at baseline (OR = .47, 95% CI = .18, 1.24, p = .1269).

### Medication use in the misdiagnosed group

Results on medication use among the 88 misdiagnosed subjects were based on 145 observations at or after a probable AD diagnosis was recorded in the NACC-UDS. Among the misdiagnosed subjects, 18.18% (16 of 88) were on a potentially inappropriate medication regimen. When using the broader definition of potentially inappropriate medication, this rate increased to 67.1% (59 of 88). Among the misdiagnosed subjects in the FTD, DLB, or cerebrovascular subgroups, 43.2% (16 of 37) were classified as being on a potentially inappropriate medication regimen. This is based on pooling the following results: a) 55.5% of misdiagnosed subjects who were identified at autopsy as having FTD (10 of 18) were treated with acetylcholinesterase inhibitors or glutamate blockers; b) 11.1% of misdiagnosed subjects who were identified at autopsy as having DLB (1 of 9) were treated with an antipsychotic medication (olanzapine); and c) 50% of misdiagnosed subjects who were identified at autopsy as having a cerebrovascular disease (5 of 10) were not treated with a statin, antiplatelet agent, or anticoagulant (the treatments considered appropriate for such conditions). The overall percentage of those in the misdiagnosed group who were treated with an anti-dementia drug at the time a probable AD was recorded in the NACC-UDS or thereafter was 64.8%.

## Discussion

This study compares characteristics of persons with an inaccurate AD diagnosis (i.e., a clinical diagnosis of AD but no neuropathological verification of AD) and an accurate AD diagnosis (those with a matching clinical and neuropathological diagnosis of AD at autopsy). Similar to other recent studies, the current analysis found that the misdiagnosed and accurately diagnosed groups significantly differed on several clinical and functional outcomes even after controlling for sociodemographic characteristics. When compared to the accurately diagnosed group, the misdiagnosed group was significantly older, less likely to be married, more likely to live alone, and more likely to have a history of cardiovascular conditions [23]. The misdiagnosed group also had a less severe illness profile in terms of dementia severity, family history of dementia, functional status and neuropsychiatric symptoms (including the presence of depression or dysphoria). This may have been due to the groups’ variation in their dementia trajectories; individuals in the accurately diagnosed group had experienced cognitive decline for approximately 1½ years longer, on average, than those in the misdiagnosed group. Similarly, a higher proportion of individuals in the accurately diagnosed group had a probable AD diagnosis at baseline than those in the misdiagnosed group. The groups did not, however, significantly differ on neuropsychological symptoms (including severity of depression) following adjustment of core sociodemographic characteristics. Interestingly, although the misdiagnosed patients were older, they had a shorter duration of symptoms and thus the onset of their decline was at 77 years of age vs. 71 years of age for accurately diagnosed patients. The younger age of onset may predispose these patients to less complicated pathology whereas the misdiagnosed, because of their older age, may be more vulnerable to other conditions (e.g., cardiovascular) [23] that can masquerade as AD. Similarly, one reason for misdiagnosis occurring among patients with cardiovascular conditions is due to multiple pathologies occurring at autopsy, which is a common occurrence even in fairly restricted clinical trial samples of AD patients [12].

Importantly, when group comparisons were adjusted for age, gender, race, marital status, education, dementia severity, time since onset of cognitive decline and whether one had a probable AD diagnosis at baseline the two groups no longer differed significantly on any clinical or functional measure except for a single depression parameter. Those misdiagnosed were less likely to have depression or dysphoria when compared to the accurately diagnosed group. This depression-related finding should be evaluated with caution, as it was based on a single item and the groups did not significantly differ on the severity of depression parameter. Considering the non-specific nature of clinical diagnosis of dementia symptoms [4], current evaluation processes of AD diagnosis may not enable clinicians to differentiate misdiagnosed and accurately diagnosed patients in routine practice when patients have similar levels of cognitive impairment, socio-demographics and time since symptom onset. This scenario would, however, be different once these patients are not of similar dementia severity level or time since symptom onset as suggested in our adjusted models of sociodemographic characteristics only (as those in the misdiagnosed group tended to have a more recent time since symptom onset as well as less severe cognitive, functional and neuropsychiatric impairment).

The findings also suggested that 18.2% of individuals in the misdiagnosed group were on a potentially inappropriate medication regimen. This percentage may be clinically meaningful as such practice could adversely and inordinately influence misdiagnosed patients’ health outcomes and lead to increased burden to patients, their caregivers, their physicians and healthcare payers. Moreover, when using a broader definition of “potentially inappropriate medication” the rate could be as high as 67.1%. The latter finding appears driven by the 64.8% of misdiagnosed patients who were treated with anti-dementia drugs. The potential personal and economic ramifications of such extensive “off label” use of anti-dementia drugs are unclear and will require future study. It is important to note that although anti-dementia drugs are indicated for the treatment of AD, treating those in the misdiagnosed group with an acetylcholinesterase inhibitor or a glutamate blocker is likely not inappropriate in all circumstances as such medications are often the practical and pragmatic therapeutic strategy for individual clinicians. Patients with non-AD dementias may also have a positive response to acetylcholinesterase inhibitors or glutamate blockers in some instances. Nonetheless, an accurate clinical/*in vivo* diagnosis is necessary in order to tailor treatment of actual underlying conditions rather than broad non-specific clinical syndromes.

The current study helps to highlight the importance of making an accurate diagnosis of AD in clinical practice. Improving diagnostic accuracy in clinical settings, and especially ruling out AD, may help reduce unnecessary treatment as well as increase the administration of appropriate therapy for patients’ conditions. There are growing efforts to improve diagnostic accuracy of AD, including the use of biomarker testing and especially biomarkers with evidence for compelling negative predictive value (i.e., when a patient’s test is negative it is most likely correct) [2,38,39]. Given the clinical complexity of distinguishing between potentially misdiagnosed and accurately diagnosed patients as demonstrated by our empirical results, biomarkers may provide a useful tool to clinicians to avoid or minimize possible misdiagnosis. Recent studies have found, for example, that the knowledge of beta amyloid positron emission tomography scan results can lead to substantial changes to clinicians’ diagnoses and intended management plans [40,41]. Such findings suggest that the use of new, more accurate diagnostic approaches may complement clinical diagnostic procedures for select patients and help improve diagnostic accuracy in clinical practice, especially decreasing the rate of false positives. The goals of the current analysis were to analyze key variations between those with accurately diagnosed AD and false positives, but in order to test the full accuracy and value of such biomarkers samples must include not only “false positives” (e.g., the misdiagnosed group in this analysis) but also false negatives to establish both sensitivity and specificity. Moreover, it is likely there are important ramifications for those who are not diagnosed with probable or possible AD but are later found to have AD-related pathology (which is often mixed). This can serve as an important focus for future descriptive and clinical research on the health and cost outcomes of misdiagnosed AD.

The results need to be evaluated in light of several study limitations. First, it is unclear whether the findings can be generalized to patients evaluated in routine clinical practice as subjects in this study were assessed at National Institute on Aging Alzheimer’s Disease Centers (ADCs) which are predominately urban, university medical centers that have recruited mostly (approximately 90%) white participants [26]. Second, the current findings on potentially inappropriate medication use were based on a small sample size and will require replication. Data on the medical rationale for the choice of treatments were not available. Third, the high proportion of participants in the NACC-UDS with a likely AD diagnosis before enrollment in the NACC-UDS, the annual frequency of follow-up assessments in the NACC-UDS, the relatively small number of available assessments after the visit in which probable AD was first recorded, and the fact that medication use was recorded in the 2 weeks prior to a NACC-UDS assessment are among the other study limitations. Infrequent assessments indicate fewer opportunities to capture use of potentially unnecessary medications, suggesting that the current findings may have underestimated the true prevalence of such pharmacological therapies (as well as capturing use of appropriate medications). Last is the extensive missing data on the Geriatric Depression Scale which led to our use of less robust depression parameters (neither of which are empirically-validated measures of depression). The statistical differences we found on the single item measure of depression were reversed for those with available GDS data (due in part to those with severe dementia not completing the GDS in the NACC-UDS). Finally, the results should be considered in light of the fact that some of the data are based on self-report without the benefit of informant information to confirm diagnosis. As individuals who lived alone or were unmarried were more likely to be misdiagnosed, clinicians may not have had the same quality of data available for these participants.

## Conclusions

This study highlights the importance of making an accurate diagnosis of AD in clinical practice (and especially ruling out AD) in order to reduce potentially inappropriate treatment for patients’ conditions. Additional research is required, however, to demonstrate the link between improved diagnostic accuracy and impaired patients’ health outcomes. A greater understanding of the empirical associations between inappropriate treatment and adverse health outcomes in misdiagnosed AD patients would advance the current state-of-the-art of clinical AD research.

## Competing interests

Joseph E. Gaugler, David L. Roth, and Tolulope Fafowora received funding from Eli Lilly and Company to complete this analysis. Haya Ascher-Svanum is an employee of Eli Lilly and Company. Andrew Siderowf is an employee of Avid Radiopharmaceuticals, Incorporated, which is a subsidiary of Eli Lilly and Company. Thomas G. Beach performs research services (payments to his research institute, not to him) as part of contractual agreements with Avid Radiopharmaceuticals/Eli Lilly Corporation, GE Healthcare, Piramal Healthcare and Navidea Biopharmaceuticals.

## Authors’ contributions

JEG had responsibility for writing, editing, and submitting the entire manuscript, oversight of data analysis, conceptualization of the study questions, and interpretation of results. HA-S had responsibility for writing and editing the manuscript, oversight of data analysis, conceptualization of the study questions and interpretation of results. DLR had primary responsibility for all data management and analysis. TF edited the manuscript and reviewed the clinical interpretation of the empirical results. AS edited the manuscript, provided input in analysis interpretation and presentation, and provided in-depth clinical expertise on inappropriate medication use in dementia. TB edited the manuscript and provided guidance on the use of his original data on misdiagnosis. All authors read and approved the final manuscript.

## Pre-publication history

The pre-publication history for this paper can be accessed here:

http://www.biomedcentral.com/1471-2318/13/137/prepub
